# Farm forests, seasonal hunger, and biomass poverty: Evidence of induced intensification from panel data in the Ethiopian Highlands

**DOI:** 10.1007/s13280-023-01954-w

**Published:** 2023-12-15

**Authors:** Nathan Morrow, Nancy B. Mock, Andrea Gatto, Andrea Colantoni, Luca Salvati

**Affiliations:** 1 Tulane University School of Public Health & Tropical Medicine, 1440 Canal Street, New Orleans, LA 70112 USA; 2 Wenzhou-Kean University, Zhejiang Province, Wenzhou, 325060 China; 3 Centre for Studies on Europe, Azerbaijan State University of Economics (UNEC), Baku, Azerbaijan; 4 Department of Agriculture and Forest Science, Università Della Tuscia, 01100 Viterbo, Italy; 5 Department of Methods and Models for Economics, Territory and Finance (MEMOTEF), Faculty of Economics, Sapienza University of Rome, Via del Castro Laurenziano 9, I-0061 Rome, Italy

**Keywords:** Food security, Food systems, Nature-based solutions, Panel data, Resilience, Sustainable intensification, I32, O13, Q01, Q16, Q18, Q23, Q56

## Abstract

Seasonal hunger is the most common food insecurity experience for millions of small dryland farmers. This study tests the relationships between food insecurity, farm forests, and biomass poverty using a longitudinal dataset from the Amhara region of Ethiopia. These data form part of the Ethiopia Socioeconomic Survey, which collected panel data over three survey rounds from 530 households between 2011 and 2016. This dataset represents a collection of unique socioeconomic, wellbeing, and micro-land use measures, including farm forests. Hierarchical mixed effect regression models assessed the relationship between food insecurity and farm forests as well as the conditional effects of biomass poverty among the poorest farmers and women-headed households. Over a six-year study period, farmers reported increased stress from smaller land holdings, higher prices, and climate-related shocks. A clear trend towards spontaneous dispersed afforestation is observed by both researchers and satellite remote sensing. Model results indicate, dedicating approximately 10% of farm area to forest reduces months of food insecurity by half. The greatest reductions in food insecurity from farm forests are reported by ultra-poor and crop residue-burning households, suggesting that biomass poverty may be a major constraint to resilient food security on these farms. This research provides novel quantitative evidence of induced intensification and food security impacts of farm management preserving and building stores of biomass value as green assets. The results reported here have important implications for nature-based solutions as a major strategy to achieve sustainable development in some contexts.

## Introduction

Globally, there are nearly one trillion trees growing outside designated forest biomes (Crowther et al. 2015). Humans have played a significant role in bringing trees into deserts, grasslands, savannas, and other ecoregions via development and agriculture. Recent estimates identifying individual trees from high-resolution satellite imagery suggest the presence of 433 million trees on African croplands (Reiner et al. 2023). As Humans have manipulated ecosystems making trade-offs for different desirable services (Kareiva et al. 2007), incorporating benefits of trees and treelike perennials appears to be synonymous with human settlement outside forests.

Trees outside of forests may act as a natural climate solution, improving the livelihoods of up to one billion people in rural areas and sequestering large amounts of carbon, but this supposition is largely unquantified (Skole et al. 2021). Research into competition for water and light resources, along with the allopathic effects of some tree species, has produced conflicting results on the relationship between the presence of trees on farms and primary crop production (Castle et al. 2021; Mallen-Cooper et al. 2022), leading to a wide range of local policies that often restrict planting certain tree species on agriculturally productive land: Specifically, eucalyptus cultivation has been banned in parts of Ethiopia (Jagger and Pender 2003; Kassa et al. 2011; Alemayehu and Melka 2022). Forestry regulations often apply to indigenous species of trees on private land, limiting management choices, restricting tree tenure, and discouraging the planting or maintenance of non-commercial species (Arvola et al. 2020) such as traditional multipurpose trees in Ethiopia (Lelamo 2021).

Satellite remote sensing analysis estimates forest cover, where at least 30% of an area is treed, for Ethiopia at 11% of the national land surface (Global Forest Watch 2023), with most of the naturally forested area confined to the southwestern regions of the country. Forests are comparatively rare in the Amhara region in the northern highlands, comprising less than 0.3% of the national tree-covered area. The region has been characterized by an agricultural mosaic dominated by grasslands and varying extents of forested area for approximately 2500 years (Darbyshire et al. 2003), with cycles of deforestation and afforestation driven by technological change, political events, and climatic factors (McCann 1997). National forest management and tree-based landscape restoration strategies have often failed to consider the complex environmental history of the Amhara region, assuming a unidirectional phenomenon in which population growth leads to deforestation by way of expanding small-scale agriculture and overharvesting biomass fuels (Ethiopian ReDD + Secretariat 2018; MEFCC 2018); even as the policy evidence base is often limited at spatial and temporal scales that are incongruous with more complex local realities (Wiegant et al. 2022). Beyond deforestation, policy aligns with authors who propose increasing population pressure as the overall driver of environmental degradation, food insecurity, and conflict that eventually led to famine conditions in Ethiopia (Haile 2004; Lemenih and Kassa 2014).

Seasonal hunger is the most common experience of food insecurity for small holder farmers in Africa (Devereux et al. 2008, 2013; Vaitla et al. 2009). Research from Ethiopia finds persistent seasonal patterns of insufficient calorie consumption (Dorosh and Minten 2020) and declines in nutritional status (Abay and Hirvonen 2017). Small holder farm households can be caught in a vicious cycle of periodic food insecurity: Low income for staple crops at harvest time is followed by high prices for those same staples in periods of greatest need before the subsequent harvest. This pattern of repetitive seasonal deprivation exhausts the potential to invest in household livelihood assets and depletes soil productivity (Chambers et al. 1981; Blaikie and Brookfield 2015/1987).

Ester Boserup, a twentieth century economist, is well known for her insights into the role of gender and innovative intensification in shaping the dynamics of population growth and food production. She provides a counterargument to the notion that a cycle of food insecurity and environmental degradation is inevitable as population density increases. Restoring fertility to farms whose holdings had become too small for long fallow periods motivated Boserup’s work on a theory of induced intensification (Boesrup 1965, p.12). She argued that agricultural intensification and improved food security was made possible by increased market demand and labor availability as population density increased. Her historical analysis found that a trend toward smaller farms tends to shift agri-food systems from extensive to more intensive cropping, suggesting that communication of innovations based on traditional knowledge plays an important role in observed systems change. At the same time, market dynamics, poor infrastructure, and misguided agricultural policy may present obstacles to innovative practices, leading to extreme poverty and alienation.

As population has increased in Ethiopia, spontaneous afforestation of small farms has become the primary source of new tree cover in the highlands (Mekonnen 2009; Lemenih and Kassa 2014; Alemayehu and Melka 2022), with authors attributing land use changes to improved tree tenure and relaxed on-farm tree restrictions. The literature documenting *induced intensification* (i.e., the conditions under which increasing population gives rise to higher levels of agricultural production) has not previously focused on traditional agri-food systems that incorporate trees. However, a recent paper by (Tong et al. 2022) suggests induced intensification as the cause for their observed association of satellite-derived cropping intensity measures and population density for three agroforestry systems in the Sahel region of Africa. The United Nations’ Food and Agriculture Organization defines food security as the outcome of an agri-food system (FAO 2021). Even as research from Ethiopian highlands indicates there are more trees where there are more people (Duriaux-Chavarría et al. 2020), tree-based induced intensification has not been extensively studied, nor has it been modeled with quantitative food security measures.

The aim of our study is to measure the dynamic association of on-farm trees with reported periodic food insecurity that may represent a carbon-centric nature-based example of Boserupian intensification of small farms in the highlands of Ethiopia. Evaluating farm-level tradeoffs between cropped and treed areas at representative regional population levels has been hampered by a lack of appropriate data; however, the Ethiopian Socioeconomic Survey (ESS) has rendered it truly tractable for the first time. This data set includes a measure of “farm forests” to describe the area of a given farm that is covered with trees but is not primarily a cropped field or orchard. The questions that guide our analysis include: (i) Is the area dedicated to trees on individual farms associated with food security or food insecurity? (ii) Do reported periods of food insecurity increase or decrease as more farm area is forested? (iii) Do area measures of farm forests add explanatory information beyond the association of food security with wealth-related consumption or asset measures? (iv) What do biomass management tradeoffs between fuel and food requirements on small Ethiopian farms reveal about the role of trees in the agri-food system?

The paper is organized as follows: The background context to this paper is presented in two parts. The first subsection, “Boserup’s stage sequence from seasonal hunger to sustainable induced intensification”, reviews theoretical aspects of Boserup’s concept of induced intensification and key extensions necessary for considering factors that contribute to decisions affecting farm management. The second subsection, “Historical patterns of deforestation and afforestation in Ethiopian Highlands", places currently observed afforestation in the context of Ethiopian history. The “Materials and Methods” section describes the food security response variable and the selection of explanatory variables with associated descriptive summary statistics, and the spatial multilevel generalized linear mixture modeling approach to the clustered longitudinal data is presented. Model results from hierarchical mixed effect regression specifically measuring the relationship between area of farm forest and periods of food insecurity are presented in the “Results” section. In the “Discussion” section, we discuss the implications of farm forests and improved on-farm biomass management for food security and implications of green assets to reduce biomass poverty and food insecurity. In the “Conclusion and policy implications” section, our conclusion summarizes the main results and next steps for research on farm forests, biomass poverty, and food security.

### Boserup’s stage sequence from seasonal hunger to sustainable induced intensification

The historical agricultural stage sequence outlined in Boserup’s (1965, p. 30) concept of induced intensification provides a simple model for both the persistence of seasonal hunger on small farms and conditions for transformation to sustainable intensification. Each stage of intensification is marked by management practices that increase the frequency of cropping. The main drivers of transformation between stages are increases in population density and the related demand for food. Pre-modern extensive agri-food systems, which rely on clearing new land or long fallow periods, shift to short fallow systems as populations stabilize and grow. Annual cereals have very short fallow periods (only a few months per year), often complicated by high input demand to maintain fertility. Farmers without robust management systems or high levels of input, especially those with monocultural annual cereal agri-food systems, can be trapped in a cycle of land degradation and seasonal hunger.

Seasonal episodes of human suffering, deprivation, and poverty represented by recurrent periods of food insecurity have persisted at least since they were first documented nearly 100 years ago (Richards and Land 1939). Chronic vulnerability is concentrated in semi-arid lands with more pronounced dry seasons (Barrett 2014), but climate itself is not the sole or even the primary cause of seasonal patterns of deprivation. This is evident in the pre-harvest lean season of tropical dry regions, when hunger counterintuitively reaches its peak during the rainiest period (Chambers et al. 1981, p.218). Measuring the average difference between high pre-harvest and lower post-harvest prices, a mean seasonal price gap of 28.3% is observed across African markets with purchasing power reduced by one third during the period of greatest need and exacerbated price gaps in the most vulnerable isolated and marginal markets (Gilbert et al. 2017). The lean season “is the time of year when poor people are at their poorest and most vulnerable to becoming poorer” (Chambers et al. 1981, p. xv). Risk of infectious disease and natural hazards such as flooding often coincide with the hungry period (Thomson et al. 2018), and productive assets are often lost or sold to cope with hardships (Chambers 1984; Krishna 2011).

Boserup (1965, p. 39) focused on structural on-farm labor dynamics with distinct peaks in demand at planting and harvest times as the primary limiting factor in these low-input non-mechanized annual cereal agri-food systems. With labor demand focused on a single planting and a single harvest, the productivity of labor averaged over the entire year is very low. Several authors have discussed this “slack season” of low labor demand (see Sen 1966; Lipton 1983; Feuerbacher et al. 2020). Fields dedicated to single-season crops spend much of any given year sitting idle with no crops being planted, harvested, or growing. The resulting low wages severely limit potential household income, increasing risk of hunger and malnutrition on small farms.

For Boserup, the pathway out of seasonal hunger is increased population with accompanying increased market demand, technological exchange, and infrastructure inducing further intensification (1965, p. 42). The eighteenth century European agricultural transformation was enabled in large part by the introduction of fodder crops to restore fertility in a multi-crop rotation. The widespread adaptation of traditional gardening practices originating in the Italian Po valley and Flanders are identified by Boserup as drivers of agricultural transformation through a process of innovation diffusion, allowing intensification for multiple harvests on small plots. She notes in Asia that innovations in irrigated systems allowed multiple crop cycles over the course of the year spread across the continent, leading to intensified production on small farms. Traditional knowledge is now widely recognized as fundamental to scaling up agroecological approaches for sustainable food system transformation worldwide (Gliessman et al. 1981; Gliessman 2016;.

Boserup’s model of induced intensification has been critiqued and expanded on for nearly six decades. Contemporary land use change modeling, which often considers farm and forest dynamics, has benefited from Boserupian formative insights on intensification, sustainability, and agri-food system transformation (Turner and Ali 1996; Turner and Fischer-Kowalski 2010). A large body of literature is dedicated to the extension of the model and adaption to specific cases with different rates of population growth; however, critiques pertaining to the role of farm management and the farmer's agency in making decisions about on-farm land use are germane to our questions about farm forests and food security. The original Boserup model simplifies farm management capacities and farmers’ agency for innovation, reducing them to reactionary responses to population pressure and trivializing the critical influence of other factors in household decision making (Brookfield 2001). Farmers' decisions are based on their specific circumstances, and the trade-offs they must make are facilitated and limited by a wide variety of individual and structural factors.

Ongoing debates around nature-based solutions to sustainable intensification, land sparing, and planetary boundaries call into question the optimistic Boserupian presumptions around indefinite agricultural intensification and the lack of attention to the intensification process’ collective impact on common resources (DeFries et al. 2012; Soby 2017). Limited on-farm resources may be related to limited access to resources in common areas, either through policy or degradation. Water and energy are major limiting factors for agriculture (D’Odorico 2018), and in many agri-food systems, availability of these resources is largely determined by access to common areas. Wood et al. (2018) found a strong association between sustained fertility of small farms and proximity to forested common areas with soil rich in organic matter. Research on induced intensification has largely ignored the effects of these transfers of biomass from common areas to the farm, and limits and restrictions to these resources, on farmer decision making. Households addressing their most significant limiting factors, such as energy and biomass, may choose to intensify with non-food producing trees rather than focusing on food crop intensity alone.

### Historical patterns of deforestation and afforestation in Ethiopian Highlands

Historical data on the extent of Ethiopia’s forestation throughout the years is scarce and unreliable; however, there is ample evidence suggesting the existence of longstanding policies and practices to manage tree resources. Fossil and lake pollen records indicate that climate was a major driver for shrubs and trees expanding to the Bale Mountain central highlands after the last glacial maximum, with juniper-podocarpus forests taking root only after BP 4500 (Umer et al. 2007). Pollen records indicate the earliest evidence of fire-related reduction of the shrub-like podocarpus in northern Ethiopia around 2500 BP and in southern Ethiopia around 1850 BP (Darbyshire et al. 2003). Concurrent with reductions of podocarpus, the first signals of anthropic afforestation through selective felling led to increases in a potentially preferred species, *Juniperos procera* (Bonnefille & Hamilton 1986). These ancient indications of afforestation efforts correspond with an eyewitness account from the sixteenth century of “reforesting mountain-sides with *tidh* trees (*Juniperus procera*)” at the order of Emperor Ya’qob (Pankhurst 1995).

Three hundred years after the reported juniper planting, Emperor Menelik II, searching for a solution to a wood fuel shortage that threatened the sustainability of the capital at Addis Abba, imported a variety of eucalyptus seedlings to be tested in a trial plantation in 1894–95 (Von Breitenbach 1961; Horvath 1968; Pukkala and Pohjonen 1990). Some seedlings thrived, and local landowners quickly recognized the value of the trees’ capacity for rapid growth and resistance to disease. Although policy has varied over the years from place to place, rapid adoption of eucalyptus was supported by tax incentives from the start, with reduced taxes for land with trees and public support for distribution of seedlings beginning at the turn of the twentieth century. These incentives mostly favored the elite, while the peasant class (and later, poor farmers) faced punitive fines or punishment unless they obtained official permission to access forest resources and harvest out-of-forest trees (Kebede 2002).

In 1975, the government of the Derg, which opposed the feudal system in Ethiopia, nationalized tree plantations and private small holder tree crops under the revolutionary Proclamation to Provide for the Public Ownership of Rural Lands (GoE 1975), and forested areas were placed under local control (Gebreselassie 2006; Crewet et al. 2008;). With uncertain land tenure and heavy restrictions on all tree harvesting, there was little incentive on the part of the public to plant or care for trees (Kassa et al. 2011). Although the causes were complex, massive droughts in 1984 and 1989 resulted in widespread famine conditions that underscored the poor resilience of annual crop and livestock–dependent livelihoods. The government later partnered with international organizations on massive reforestation campaigns, but the majority of these common woodlots and forests were cut down during the political transition of 1990–1991, leaving an estimated forest cover of less than 3% in Ethiopia, concentrated in the southwest (Kebede 2006).

When the prohibition on tree cutting was rescinded in 1991, there was a boom in on-farm tree planting to meet construction and fuel wood demands, with more than 90% of the country’s rural energy needs met with biomass (Ethiopian Ministry of Water and Energy 2013). According to Turnbull and Booth (2002, p. 55), “many people in Ethiopia are absolutely dependent on eucalyptus as a source of fuel and house building material.” Planted forest area, defined as continuous tree cover of more than 0.5 hectares without agricultural or other use, quadrupled in Ethiopia between 1990 and 2020, while naturally regenerating forest area declined by approximately 15% (FAO 2020).

**Fig. 1:**
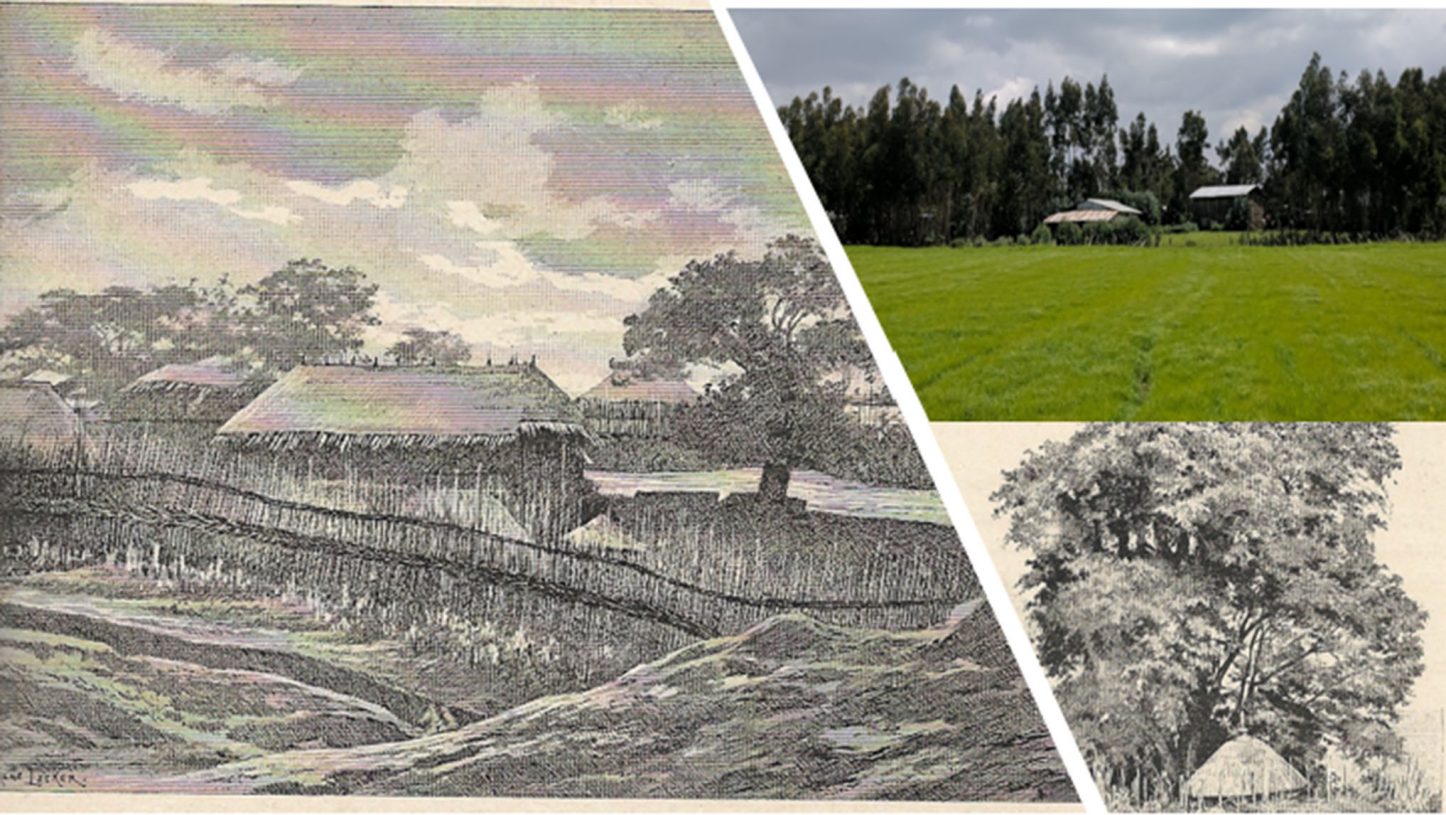
Comparison of farm forest sketches from 1882 and photo taken in 2018 (photo credit: Nathan Morrow)

Capturing scenes of deforestation and reforestation in a travel journal, a Jesuit priest named Jules Borrelli (1890) visited Ethiopia as part of an official Vatican mission to the Abyssinian empire. His sketches of the present-day Amhara region in the 1880s depict trees growing on farms and are strikingly similar to contemporary images of farm forests (Fig. 1).

**Fig. 2:**
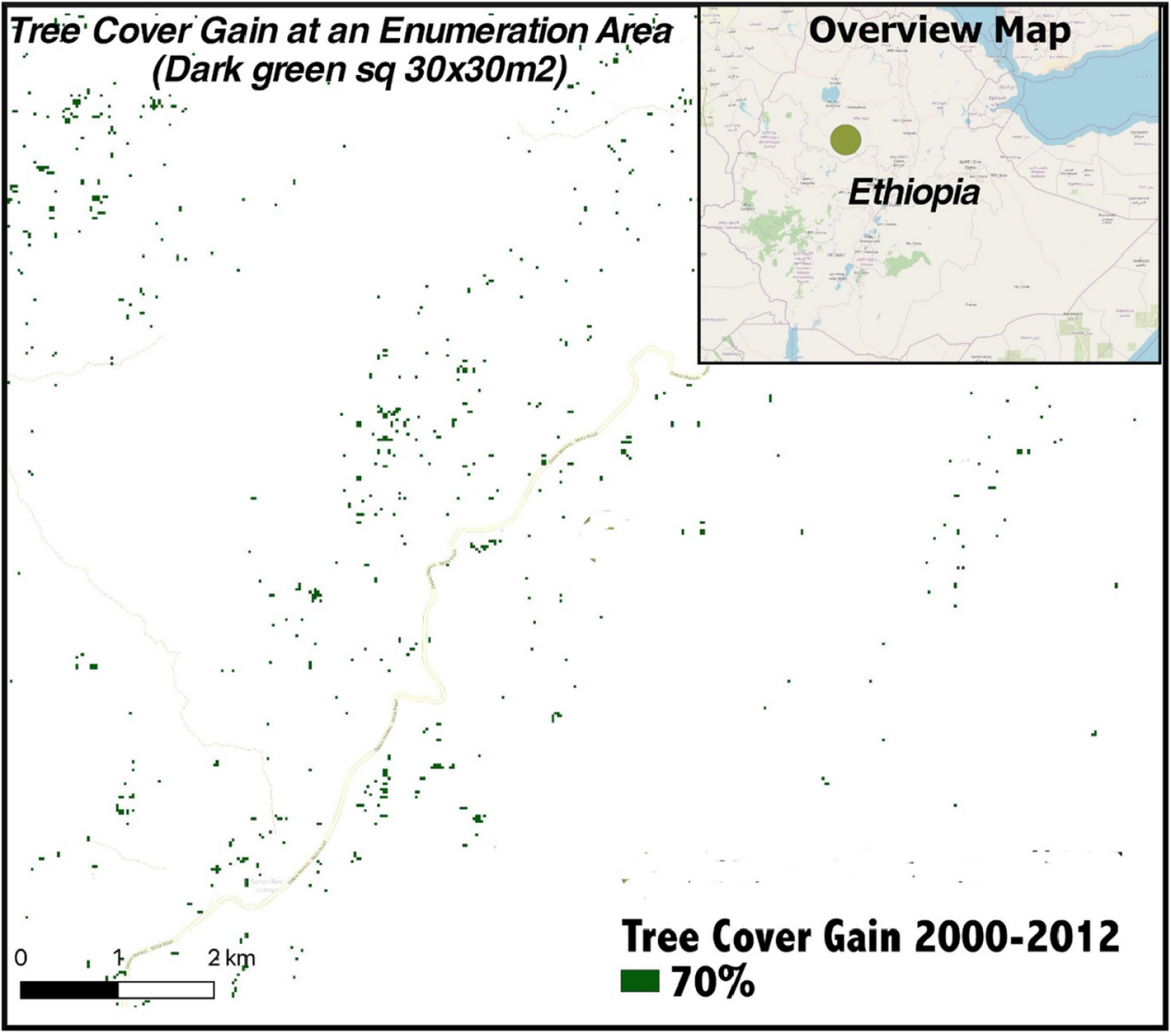
Satellite-derived tree cover gain from 2000–2012 around ESS Enumeration Area #207 from Hansen et al. (2013) with background © OpenStreetMap contributors (2021)

Teketay et al. (2010) describe the well-established and expanding practice of farm forestry in the rural highlands around the turn of the twenty-first century. In recent decades, reduced regulation and improved tree-tenure have led to policy shifts in Ethiopia to promote increased private tree planting and preservation (GoE 2011). The subsequent expansion of eucalyptus and other trees across the landscape was immediate and highly visible (Dercon and Ayalew 2007); in fact, it was observable from space. Hansen et al. (2013) mapped global tree cover gain from 2000–2012 and identified 30 × 30 square-meter areas of earth that went from untreed to at least seventy percent tree cover within this 12-year period. As seen in the scattered distribution of dark green squares in Fig. 2, satellite observations confirm the presence of hundreds if not thousands of small farm forests and other afforesting areas.

Eucalyptus tree planting on farmland remains controversial. Regional and local policy is inconsistent and reflects conflicting scientific findings on eucalyptus’ allelopathic effects and toxicity to other plants. For instance, eucalyptus seems to be deadly to nearby corn crops (Baumer 1990) but has little or no effect on millet or sorghum (Chanie et al. 2013). In real-world conditions, eucalyptus’ direct impact on food crops is generally considered to be manageable and potentially beneficial to weed and pest control (Singh et al. 2001; Zerfu 2002). Literature on the livelihood and economic benefits of on-farm trees are more common for study sites on real-world farms in communities with demand for eucalyptus products (e.g. Mekonnen et al. 2007; Ango et al. 2014; Eshetu et al. 2018)).

Ethiopia has more than 400 endemic trees and shrubs (Vivero et al. 2005). Poschen (1986) found that Ethiopian farmers had clear preferences for different trees in different sampled water catchments with site-specific pollarding regimes to protect crops. Wanza (*Cordia africana*) is preferred among maize-growing farmers and has been shown to improve soil traits (Yadessa et al. 2001) and, when ingested, is helpful in treating diarrhea (Giday et al. 2016). The common buckthorn gesho (*Rhamnus prinoids*) adds a hops-like flavor to the beer-like fermented beverage *tella*, sales of which offer an important source of income in rural areas, particularly for women (Lee et al. 2015). Birbira (*Milletia ferruginea*) has a variety of uses, most notably as a poison used to intoxicate and then catch fish. Woriya (wild olive trees) are also found on farms in Ethiopia and were likely first used for their excellent fuel characteristics before being domesticated for oil production in the Mediterranean area around 6500 BP (Aerts et al. 1997; Kostelenos and Kiritsakis 2017). These and other trees thrive in Ethiopian farm forests to serve a wide variety of household needs.

## Materials and methods

The Ethiopian Socio-Economic Survey (ESS) collected three waves of panel data in 2011–2012, 2013–2014, and 2015–2016 providing demographic, socioeconomic, and livelihood statistics as part of the World Bank Living Standards Measurement Survey Integrated Survey on Agriculture program (Central Statistics Agency of Ethiopia and World Bank Group 2017). The data used for the current study is available from the World Bank Microdata site (https://microdata.worldbank.org, accessed January 15th, 2023) and is accompanied by extensive documentation of each survey campaign, survey instruments, and detailed information about the consumption indicators. The dataset is representative at the national and regional levels. Our study focuses on Amhara region. Data cleaning resulted in a balanced panel of 530 households in 61 enumeration areas.

Variables were selected with the aim of maximizing parsimony of the model while including common food security measures and proxies indicated by survey respondents themselves as primary causes of food insecurity. From the food security module of the ESS, Months of Food Insecurity (MIns) was most closely associated with periodic seasonal hunger and was therefore selected as the response variable. ESS contains detailed plot-level data describing the specific uses of areas of a given farm. Our primary explanatory variable of interest is Farm Forest Area (FFArea), a description of land use distinct from orchards or crop fields with trees. Wealth, represented by typical assets and a consumption measure, is included as an explanatory variable. Explanatory variables for women-headed households and indicators of biomass management are also included.

### Descriptive statistics for response variable, primary explanatory variable, and explanatory covariates

Here, we present basic descriptive statistics for response variable *Months of Food Insecurity* (response variable: MIns). MIns is a typical microeconomic indicator in areas with seasonal hunger: Survey participants are asked to indicate which months in the previous year presented “a situation when you did not have enough food to feed the household.” As implemented in the ESS, MIns is a slight adaptation of the food security indicator "months of adequate household food provision" included in post-harvest agricultural and food aid project assessments focused on household cereal stores (Bilinsky and Swindale 2010). MIns includes stored cereal as well as food purchased on the market, gathered, or received in-kind and is a goal level indicator for the Government of Ethiopia’s operational plan for the Alliance For a Green Revolution in Africa (GoE ATA, n.d.). MIns saw a peak in Wave 2 of the ESS with a mean of 1.05 months or 32 days of reported food insecurity (see Table 1). Wave 2 also represented the highest number of households, with more than one third of all households surveyed reporting one or more months of food insecurity.

**Table 1:** Months of Food Insecurity (MIns) measured in 3-waves of ESS survey

	Mean	Standard Error	HH reporting# MIns
MIns Wave 1	0.79 (24-days)	0.07	135 (26%)
MIns Wave 2	1.05 (32 days)	0.07	188 (36%)
MIns Wave 3	0.48 (15-days)	0.06	92 (17%)
#Obs = 530			

*Farm Forest Area* (explanatory variable: FFArea) as measured in hectares is a primary explanatory variable in our multilevel longitudinal model. The ESS measured the area of land use for each purpose and each combination of crops as a distinct household-managed plot. FFArea is the total area of plot(s) where the land use was classified as “forest” (as opposed to “orchard” or “pasture”). Data regarding specific kinds/species of trees on farm forests is missing, but Wave 2 field notes for some plots indicate that some areas classified as farm forest included eucalyptus. Fruit trees, or commercial crops from woody shrubs like coffee that are intercropped or planted as small orchards, are measured separately and classified as *cultivated land use*. In the six years between the first ESS survey visit and the end of the third wave of measurements, the mean FFarea for the Amhara Region increased from 830 m^2^ (0.08 hm^2^) to 1261 m^2^ (0.13 hm^2^) for areas with farm forests (see Table 2). A repeated t-test of the means between Wave 1 and Wave 3 found that the increase in area was statistically significant at p ≤ 0.001. The smaller FFarea increases from Wave 1 to Wave 2 and Wave 2 to Wave 3 were also significant at p ≤ 0.01 each. The number of households reporting FFarea also increased (n = 110 in wave 1 to n = 177 in wave 3). FFarea and total farmed area were significantly corelated at p ≤ 0.05 only in Wave 3. Mean total farmed area size did not reflect any statistically significant change between waves according to t-test or Wilcoxon rank sum test.

**Table 2:** Farm forest and total farm area measured in 3-waves of ESS

	HH reporting farm forests	Farm forest area, mean hm^2^ (SE)	Total field area, mean hm^2^ (SE)	Pearson Correlation of total farm/farm forest area *(p ≤ 0.01)
Wave 1	110	0.08 (0.02)	1.34 (0.04)	0.11
Wave 2	155	0.1 (0.01)	1.39 (0.04)	0.15
Wave 3	177	0.13 (0.02)	1.27 (0.04)	0.38*

The ESS data set included a question about the causal factors driving food insecure periods that informed our choice of covariate explanatory variables. Small land size, high food prices, drought, pests, floods, and inadequate agriculture inputs or tools were reported as causes for extended periods of food insecurity by 396 households over the three survey waves. Small land size was the most frequently reported cause (70% of households). The second most frequently reported cause was high food prices (37% of households). The survey allowed households to identify up to three different causes, and we found that the most common combination of causes was small land and high prices (24% of households). In combination with small land size or high prices, drought and lack of agricultural inputs were also reported by 32% and 26% of households, respectively. A relatively small percentage of households, 18%, identified shocks of drought, plant pests, or floods without small land size or prices as drivers of MIns.

Building on the ESS household reports showing the importance of structural factors as the primary causes of food insecure periods and their consistency with explanations for hungry periods from the academic literature, we include explanatory covariates in the model to control for household or location-specific differences in demographics, wealth, and biomass management. Food insecurity and poverty is more prevalent in Ethiopian households headed by women (Negesse et al. 2020), and this is included as binary explanatory variable in the model (explanatory covariate #1: FHH). *Per capita consumption* (explanatory covariate #2: Cons) is a continuous explanatory variable provided by the Ethiopian Central Statistics Agency and the World Bank (CSA and WB 2017) with adjustments for between-wave inflation and regional price differences to yield the per-person value of everything purchased or produced by the households for members’ own use measured in one thousand Birr units.

Three asset-based measures of wealth are included as explanatory covariates to better isolate any additional role of FFArea in reducing periods of food insecurity. Livestock is a common asset for rural Ethiopian households and is measured by the unidimensional variable Tropical Livestock Units (explanatory covariate #3: TLU) using a set of conversions provided by the Food and Agriculture Organization (FAO 2023). To create unidimensional asset measures that can be compared over time in a longitudinal analysis, count data from socioeconomic surveys must be transformed (Naschold 2012; Barrett et al. 2016). We used factor analysis to reduce dimensionality on ESS data. To create an index of household objects, we selected nine surveyed items that were reported by at least 5% of households; 75% of the variance was explained by the first factor that weighted positively on all items. We simply used the scores of this first factor to weigh our asset wealth variable based on household objects (explanatory covariate #4: HHObjs). For the four agricultural implements owned by at least 5% of households, a single factor explained 98% of the variance. We used these scores to weigh an additional asset wealth variable based on agricultural implements (explanatory covariate #5: AgImps). A binary variable for households that used crop residue or dung as a primary fuel source was included to capture biomass management practices of those households using less preferred fuels (explanatory covariate #6: CresDung).

Months of Food Insecurity were significantly correlated with all explanatory variables except consumption and households whose primary source of fuel was crop residue or dung (see Table 3). Area of farm forests was significantly and negatively correlated with months of food insecurity and women-headed households. Area of farm forests was significantly and positively correlated with all other explanatory variables. Women-headed households were significantly and negatively correlated with all explanatory variables except months of food insecurity and area of farm forests. Overall, correlations were significant but weak considering the sample size, although larger farms were more strongly associated with having more agricultural inputs and tools.

**Table 3:** Spearman non-parametric cograduation coefficients for response and explanatory variables

	MIns	FFArea	FarmSize	HHObjs	AGImp	FHH	Cons	TLU
FFArea	− 0.16*							
FarmSize	− 0.14*	0.21*						
HHObjs	− 0.26*	0.28*	0.26*					
AGImp	− 0.18*	0.21*	0.54*	0.40*				
FHH	0.13*	− 0.1*	− 0.38*	− 0.21*	− 0.42*			
Cons	− 0.06	0.07*	0.02	0.17*	0.06	0.02		
TLU	− 0.21*	0.22*	0.56*	0.33*	0.45*	− 0.33*	0.03	
CresDung	0.03	0.15*	− 0.03	0.08*	0.03	0.04	0.02	0.03

Statistical significance tested at *p ≤ 0.01, after Bonferroni correction for multiple comparisons

### Model specification

To estimate the association of farm forest area on our response food insecurity measure, the variable FFarea was added along with other explanatory variables to a three-level generalized linear mixed model. Measurements were repeated across three waves of the Ethiopian LSMS-ISA survey for 530 households (_*i*_) within 61 enumeration areas*(*_*j*_). The three-level variance components model improved estimation by modeling the sequential nesting of observations in households and households in enumerations areas. This approach includes a random variance component at each level of the nested panel structure of the data to ensure that standard errors are not underestimated, test statistics accurately reflect strength of associations, and parameters are estimated efficiently (Wooldridge 2010). Initial data analysis confirmed high levels of variability by location for both the response and explanatory variables that generally exceeded the variability between individual households in the same place; therefore, the place-based context of communities is represented by allowing a random intercept at each of the enumeration districts.

Mins, as the response variable of interest, was regressed on FFArea and the other explanatory variables with the three-level variance components model for each panel survey wave (t), household (i), and enumeration area (j) following Baltagi (2008) and Rabe-Hesketh and Skrondal (2008). The hierarchical mixture model approach allows between- and within-subject variance to be modeled separately as random intercepts and random slope coefficients (Zambon et al. 2018). In our model, household variance, as well as variability by location and location-over-wave, are modeled with separate random effects that in turn capture the high levels of local contextual and temporal variability (Bajocco et al. 2015). Our models follow a structure wherein the response variable of interest $({y})$, values for MIns, are regressed on explanatory variables and the random components as:

$$\begin{gathered} y_{{ijt}} = ~\beta _{1} + ~\beta _{2} FFarea_{{1ijt}} + \beta _{3} FarmSize_{{2jt}} + \beta _{4} HHObjs_{{3ijt}} \hfill \\ \;\;\;\;\;\; + \beta _{5} Ag\text{Im} ps_{{4ijt}} + \beta _{6} FHH_{{5ijt}} + \beta _{7} Cons_{{6ijt}} + ~\beta _{8} TLU_{{7ijt}} + \beta _{9} CresDung_{{8ijt}} + \zeta _{t}^{2} + \zeta _{t}^{3} + \in _{{ijt}} \hfill \\ \end{gathered}$$

where: *y*_*ijt*_ is the response variable for food security status Mins, *β*_1_ is a constant offset, *β*_2–9_ are explanatory variable coefficients, $\zeta _{t}^{{\left( 2 \right)}}$ is a random intercept coefficient at household level $\zeta _{t}^{{\left( 3 \right)}}$is a random intercept coefficient at location level, $\epsilon _{ijt}$ is the observation level error component.

The model uses a generalized linear model with a negative binomial link. **ζ **_**1**_ random intercept has a mean of zero and variance ***ψ***^(3)^ given covariates $\mathcal{x}$. Random slope ${\zeta }_{2k}^{(3)}{\mathfrak{t}}_{it}$ also has a mean of zero and covariance matrix Ψ ^(3)^ given covariates $\mathcal{x}$. The ${\epsilon }_{ijt}$ error term has a mean of zero and variance θ. The Generalized Linear Mixed Effect models were run using STATA 15 command library (StataCorp 2021, p.86). MINS is a count variable (0–12) with overdispersed variance that was modeled with a negative binomial distribution as:

$$log\left\{\mathrm{\rm E}\left(\upgamma |\upmu \right)\right\}= \mathrm{\rm X}\beta +\mathrm{\rm Z}\mu , \gamma \quad\sim nbinomial$$

where $\mathrm{\rm X}\beta +\mathrm{\rm Z}\mu$ is the linear predictor describing the covariate matrix and the random intercepts in matrix notation. $\mathrm{\rm X}$ is the covariate matrix for fixed effects $\beta$ which is “analogous to the linear predictor from a standard OLS regression model with β being the regression coefficients to be estimated” (StataCorp 2021, p. 9). Similarly, $\mathrm{\rm Z}$ is the covariate matrix for random effects $\mu$.

## Results

Here, we present results from the hierarchical mixed effect regression to measure the association between area of farm forest and period of food security while controlling for effects of other explanatory variables. We find area of farm forest was strongly, significantly, and negatively associated with the reported length of food insecurity as measured by MIns (see Table 4). Three additional explanatory variables were also significant at *p* ≤ 0.05 level: burning crop residue or dung, wealth measured with household objects, and livestock ownership measured as TLU. Regression coefficients for agricultural assets, per capita consumption, and women-headed households were not significant. Overall, the standard errors are low for the significant explanatory variables.

**Table 4:** Regression coefficients and standard errors from the longitudinal multilevel model

	Farm forest area (hm^2^)	Farm size (hm^2^)	Burn crop res/dung	FHH	HH Objs	Ag Imp	TLU	Cons (‘000 Birr)	Constant
Coefficient	− 2.04*	0.002	0.4*	0.19	− 0.24*	− 0.19	− 0.21*	− 0.01	− 0.29
Standard error	1.05	0.09	0.2	0.15	0.09	0.12	0.04	0.02	0.19

Statistical significance tested at *p ≤ 0.05

The likelihood ratio test indicates that the multilevel model fits the data better than a model without random components for location and household (see Table 5). The Wald statistic is the preferred goodness of fit test for multilevel models, and our strong and significant Wald statistics indicate that the explanatory variables add information to the model in a meaningful way. To understand whether farm forest area (as the primary explanatory variable) independently improved the model fit, we calculated the Wald statistic for a restricted model with only the explanatory covariates. The difference in Wald statistics, 90.03 for full model versus 87.32 for the restricted model, is greater than the standard confidence interval of 1.96, confirming the significant association of farm forest area with reduced MIns. Additionally, an Akaike’s Information Criterion (AIC) test confirmed a better fit with a lower value for the full model. A large and negative log-likelihood of − 1627 indicates general efficiency in the estimated model coefficients. The location random coefficient, estimated at 0.53, is more highly associated with the variability in MIns than the household variable component of the model.

**Table 5:** Goodness of fit statistics and random components from the longitudinal multilevel model

	Likelihood ratio-test	Wald statistic	Akaike’s Information Criterion	Log-Likelihood	Location random coefficient	Household random coefficient
	78.91*	90.03*	3276	− 1627	0.53	0.25

Statistical significance tested at *p ≤ 0.05

The marginal effect of burning crop residue or dung, estimated by holding all other variables fixed, is associated with a marginally significant increase in MIns at a $\mathrm{p}>{\mathrm{\rm X}}^{2}$ 0.09 level (see Table 6). Households have an average 37.95% increased likelihood of reporting a month of food insecurity if they burn crop residue or dung as their primary source of fuel. The predicted increase in likelihood of reporting MIns at a $\mathrm{p}>{\mathrm{\rm X}}^{2}$ 0.26 level for women-headed households was not significant in this model.

**Table 6:** Pooled marginal effect of burning crop residue or dung on MIns

	Average increased likelihood of MIns (%)	Standard error (%)	Confidence interval (%)	${\mathrm{\rm X}}^{2}$	$\mathrm{p}>{\mathrm{\rm X}}^{2}$
Burn crop residue or dung	37.95	22.31	− 5.77 to 81.68	2.89	0.09
Female headed household	16.51	15.81	− 2.82 to 59.18	1.26	0.26

## Discussion

These results demonstrate that larger farm forests are associated with shorter periods of food insecurity. They contribute to the discussion of sustainable induced intensification and to a growing body of literature linking treed areas to improved food security. We show empirical evidence for the dynamic linkage between biomass management on small farms, exacerbated by burning non-preferred fuels or ameliorated by farm forests, and a standard food security outcome measure. Studies linking positive food security outcomes and farm location proximity to natural forests emphasize the advantages of social institutions governing common access to natural capital, with a focus on livelihood strategies using forest products (Reardon and Vosti 1995; Agrawal 2001; Babulo et al. 2009; Olstrom 2009). Forests are also sources of foods and products that may improve child nutrition (Powell et al. 2011; Johnson et al. 2013; Ickowitz et al. 2014; Galway et al. 2018). However, our results support a different, emergent explanation for positive food security outcomes from indirect contributions from trees through the flow of biomass to support overall farm system function and sustainable nutrient cycling from common areas (Baudron et al. 2017; Reed et al. 2017; Wood et al. 2018), on-farm trees (Morrow et al. 2018), healthier livestock production (Wilson et al. 1980), or additional fuel for cooking nutrient-rich food such as legumes (Galway et al. 2018). As an important component of both organic sustainable farming and fuel for meeting household energy needs, biomass from treed areas both on and off the farm form a functional foundation for resilient small farms. Lack of trees may present a significant limitation to biomass cycling for sustainable maintenance of small farm fertility, particularly in regions like Amhara where common treed areas are scarce.

Although 70% of households report small farm size as a main cause of food insecurity in our study, farmers choosing management strategies that dedicate space to both food producing cropped areas and non-food producing forest areas are more successful at maintaining food security. We show that an increase in farm forest area by one standard deviation results in a reduction of approximately seven days of food insecurity, a 22–47% reduction (depending on survey wave). The mean farm forest size in our sample is less than 10% of the total mean farm area, so small areas of land dedicated to trees appears to return large food security benefits. Chambers and Longhurst (1986) first proposed that multi-use multi-variety trees on privately owned land can reduce seasonal deprivation linked to single growing seasons. The mechanism proposed was that the trees allowed access to subterranean water to produce biomass in a variety of forms to meet different household needs throughout the year. Lack of year-round biomass availability to meet farm household needs is a form of biomass poverty. We observe in Amhara that households using non-preferred fuels (dung and crop residue) report 5–11 more food insecurity days per year. Burning these forms of biomass as fuel has been proposed as an indicator of biomass poverty, since there are more efficient and beneficial uses as compost or fodder for these products on farms (Morrow et al. 2018). Although a small number of studies documented trees on farms buffering shocks as a type of “green bank account" (Leakey 2010; Mbow et al. 2014), all-purpose use of intensified tree-based biomass remains poorly understood (Garrity 2004; Garrity et al. 2010).

We found a weak but surprising association between per capita consumption and women-headed households, and limited association of both with duration of food insecurity. This may be explained by the varying recall periods of the food insecurity and consumption questions. Months of food insecurity had a 12-month recall period, whereas consumption measures concerned the week prior to the survey interview, and the survey was not conducted during the most food insecure time of the year immediately before harvest. A majority of studies find women-headed households in Ethiopia are less food secure (Negesse et al. 2020), have less land (Melesse et al. 2018), and are poorer with fewer agricultural and other assets than male-headed households (CSA and World Bank 2020). This analysis includes many of the pathway variables, such as less income or fewer assets, through which gender operates to increase food poverty, so the explanatory power of a household headed by either gender as an explanatory indicator was reduced.

The six-year ESS dataset shows an increase in households planting farm forests; in fact, farm forest expansion was significant from wave to wave throughout the survey. An intensification of the agri-food system with land use choices that are productive throughout the year, such as a farm forest, may increase total biomass production and reduce seasonal biomass production variability. Compared to more conventional measures of household, agricultural, or livestock assets, farm forests have a stronger association with reduced periods of food insecurity in the Amhara region. This may be attributed not only to increased total production of biomass, but also the flexible timing of harvest and counter-season productivity of on-farm trees when annual cereal plots are fallow. A study from the Southern Nations, Nationalities, and Peoples region in the southern Ethiopian highlands found similar benefits from flexible harvest of tree-like perennials associated with positive food security outcomes (Morrow et al. 2023).

In an era of increased interest in agroecological (Kuyah et al. 2021) approaches to sustainable intensification, a clear understanding of the mechanisms and contextual variation involved is far from complete. The function, impact, and basic areal extent of different traditional and recently intensified agri-food systems (with trees) are largely unmeasured. Although some authors, including Gleissman (1981, 2016), have recognized the importance of traditional knowledge for successful agroforestry intensification, much of the body of research comes from research stations (e.g., experimental farms) rather than direct communication with small farmers and firsthand accounts of their strategies. The editorial board for *Nature* (2020) recently concluded that insufficient research on small farms in real-world conditions limits the formulation of policies and interventions to end hunger.

Policy approaches to increase tree cover generally are increasingly well understood. Randomized control trials from Benin find that secure land tenure is a key factor in reducing deforestation (Wren-Lewis et al. 2020). Security of tenure was also associated with increased tree planting on farms in Ethiopia (Dercon and Ayalew 2007). Policies to expand tree cover are supported by research conducted by Jagger and Pender (2003) who were among the first to document that the allopathic traits of the eucalyptus in real-world settings had little impact on crop yields, in contrast to findings from controlled experiments. Iiyama et al. (2017) also found farmer-led extension that supported hyperlocalization and diversity rather than more generic agroforestry models was more effective in improving sustainable livelihood outcomes.

Farms can play an active role in afforestation rather than drive deforestation, particularly in Africa (Fairhead and Leach 1996). Results of our study of Amharan farm forests provide an example of induced or spontaneous intensification, based in traditional agricultural knowledge, that may have been triggered by reduced restrictions and improved tree tenure. Farm forestry and agroforestry-related policy should be informed by representative research in real-world community and food systems rather than findings from model farms (see review by Coe et al. 2014). Focusing on farmer agency and facilitating communication of contextually relevant innovation are promising pathways to support sustainable intensification in agri-food system transformation.

The disruption of seasonal weather patterns caused by climate change is projected to have devastating effects on annual cereal production (Shukla et al. 2019). Climate-resilient seasonality-aware agricultural intensification will be key to both meeting the needs of the growing population and stopping further environmental damage. Ensuring basic food security to an unprecedented number of humans will require shifting focus to the development of resilient agriculture that works in concert with natural systems (Rockström et al. 2017) and is informed by farmers’ knowledge of local agri-food systems (Haraway 2016; Leach et al. 2020). There is great potential for sustainable approaches to land sparing through locally determined intensification as a means to limit further destruction of forested and other common areas and help to mitigate threats to life-preserving systems at the planetary level (Lamb et al. 2016). Zomer et al. (2016) conclude that significant tree cover estimated at 10% of farm area globally is an undervalued spontaneous contribution, often of autochthonous food systems, to climate change-related carbon policy goals. Re-afforestation of farms, particularly those in communities with cultural roots in such a practice, presents a compelling nature-based pathway toward the sustainable wellbeing of both people and the planet.

This study has a few important limitations to consider. Analysis is based on secondary data from a household survey program developed to measure socioeconomic variables and conventional crop and livestock production; thus, pertinent information like farm forest tree types and variable planting or pollarding practices was not collected. Data on biomass use and management was limited and insufficient to clarify the mechanisms underlying farm-level tradeoffs and farm-specific causal pathways that connect trees to wellbeing, resilience, and food security outcomes. The study is also limited to a time period of six years. Measuring and understanding significant trends in land use, such as changes in overall farm size and planting patterns, may take decades rather than years. Since trees grow relatively slowly, panels collected over longer periods would provide greater insight into the sustainability and long-term advantages of farm forest strategies.

## Conclusion and policy implications

The highlands of Ethiopia are being afforested by individual farm households that establish and care for small private farm forests. Freed from prohibitions on tree ownership and tree felling, farmers are intensifying biomass production and storage in the form of on-farm trees as an additional asset. The results of this study demonstrate that shorter periods of food insecurity are associated with larger on-farm forests. Farm households in the Amhara region that cultivate these farm forests are significantly more likely to be food secure, especially the ultra-poor. Forested area per household correlates with differences in the expected period of food insecurity beyond conventional indicators of household, agricultural, and livestock assets. Although they occupy only around 10% of the total farm area, farm forests are associated with relatively large improvements in food security. An increase of one standard deviation in area of farm forest can reduce period of insecurity by nearly half.

Intensification of small farms based on traditional agri-food system knowledge supports Boserup’s theory of induced intensification. Ethiopia’s growing population and economy may be driving small farmers to reconsider their farm and biomass management choices, leading to an agri-food system transformation. Since non-preferred fuel choices of crop residue and dung are associated with poor food security, and since improved food security outcomes correlate with on-farm trees, biomass poverty appears to be a limiting factor for small farms. Biomass poverty may be exacerbated in areas without proximity to common area forests or pasture. The study also confirms the necessity of important adaptations to Boserup’s theory to include an expanded focus on farmer agency, biomass management, and policy context.

Successful evidence-based policy for agri-food system transformation should build on locally relevant innovation based in traditional or place-based knowledge and allow farmers agency in making decisions regarding biomass management. These approaches have the greatest opportunity for success and will likely lead to improved compliance with implementation.

Further research should validate these findings in other low-income settings in areas where seasonal food insecurity negatively impacts small landholders, mining available panel data and incorporating primary data informed by direct interaction with farmers regarding their green assets and decision-making. In-depth studies are needed to clarify drivers or limitations of expanding treed areas on farms, the perceived benefits, and the associated intensification strategies. Remote sensing data should be integrated with other socioeconomic data to further explore the spatial and temporal dynamics of afforestation on small farms.
